# Decoding autoimmune disease with single-cell immune repertoire and transcriptome sequencing: mechanisms and therapeutic opportunities

**DOI:** 10.3389/fimmu.2026.1827384

**Published:** 2026-06-16

**Authors:** Shuqing Wang, Zhao Dong, Yuefang Liu, Xianhe Ren, Xin Wang, Hongsong Yu

**Affiliations:** 1 Institute of Basic Medical Sciences, Special Key Laboratory of Gene Detection and Therapy of Guizhou Province, Zunyi Medical University, Zunyi, China; 2 Department of Immunology, Key Laboratory of Cancer Prevention and Treatment of Guizhou Province, Zunyi Medical University, Zunyi, China

**Keywords:** autoimmune diseases, immunotherapy, mechanisms, single-cell TCR/BCR sequencing, single-cell transcriptome sequencing

## Abstract

Autoimmune diseases result from a breakdown of immune tolerance, driving the aberrant activation and clonal expansion of self-reactive T and B cells. Progress has been limited by the inability to link clonal identity to functional states at single-cell resolution. Single-cell T- and B-cell receptor sequencing (scTCR/BCR-seq) now bridges this gap by simultaneously recovering paired TCR (α/β) and BCR (heavy/light) chains and gene-expression profiles, enabling direct coupling of clonotypes to cellular function and tissue trafficking. This shift from population-level averaging to clonal-resolution analysis, particularly when integrated with multi-omics, has yielded substantial insights. Mechanistically, scTCR/BCR-seq identifies tissue-homing, putatively pathogenic clones and resolves somatic hypermutation, class-switch recombination, and T-B cell interaction networks, thereby delineating the clonal basis of disease heterogeneity. Clinically, it enables tracking of clonal dynamics to inform prognosis and to predict responses to therapies such as anti-CD20 or BAFF-targeted agents. For therapeutic development, reconstructing autoantibodies from BCR clones and mapping epitopes through TCR analysis provide a foundation for antigen-specific tolerance strategies. This review synthesizes scTCR/BCR-seq methodologies and systematically charts these advances and discusses current challenges and future directions toward precision subtyping, biomarker development, and novel immunotherapies in autoimmune disease.

## 1 Introduction

The adaptive immune response is governed by the coordinated interactions between T cells and B cells. Cellular immunity is initiated when T cells recognize antigenic peptides presented by antigen-presenting cells (APCs) in the context of major histocompatibility complex (MHC) molecules via their T cell receptors (TCRs), triggering activation and differentiation into effector subsets (1–3). In humoral immunity, B cells directly recognize antigens through their B cell receptors (BCRs) and, with T-cell help, differentiate into antibody-secreting plasma cells (4). The specificity and potency of an immune response are directly determined by the characteristics and clonal dynamics of these receptors, which establishes TCRs and BCRs as central targets in immunological research. This central role is underscored in autoimmunity, where the aberrant selection and activation of self-reactive TCR and BCR clones initiate tissue injury.

The clinical manifestation of such immune dysregulation is a wide spectrum of autoimmune diseases, which are characterized by the immune system’s loss of self-tolerance, leading to inflammation and damage of specific tissues or systemic organs (5, 6). They can be broadly categorized into organ-specific (e.g., type 1 diabetes, multiple sclerosis) and systemic (e.g., systemic lupus erythematosus, rheumatoid arthritis) forms, collectively affecting a substantial portion of the global population with significant morbidity and healthcare burden (7). Current therapeutic strategies, including broad immunosuppressants and biologics targeting general immune pathways (e.g., anti-cytokine agents, B-cell depletion), have improved outcomes but often lack specificity, exhibit variable efficacy, and may incur substantial side effects. A fundamental challenge lies in the profound inter-individual heterogeneity and clonal complexity of autoimmune responses, where a diverse repertoire of self-reactive lymphocyte clones drives pathology.

**Figure 1:**
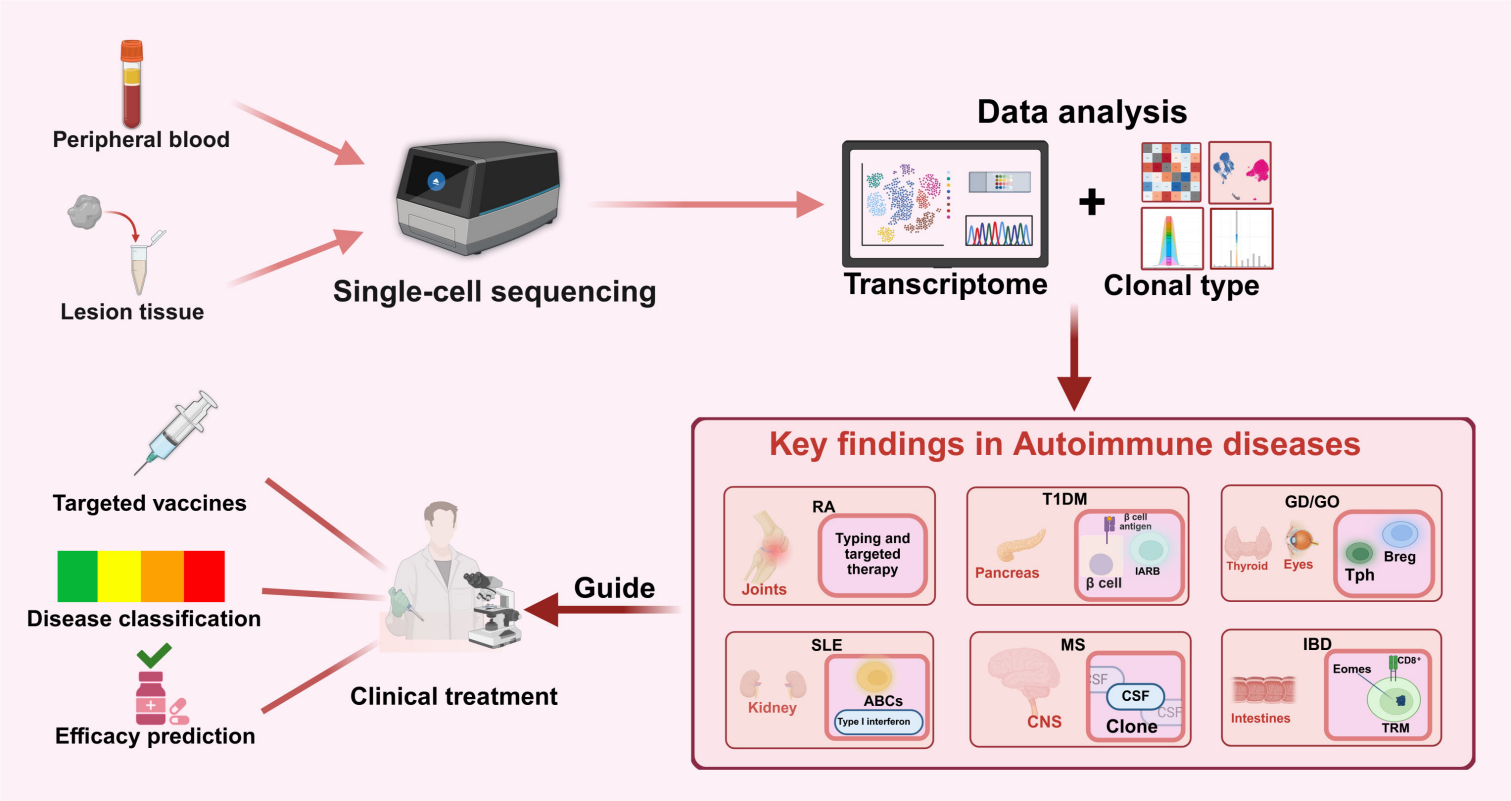
Analytical and translational framework integrating scTCR/BCR-seq with scRNA-seq in autoimmune disease research. This figure presents the workflow from sample collection to clinical application. Peripheral blood and lesional tissues are subjected to single-cell sequencing to simultaneously capture transcriptomes and paired TCR/BCR clonotypes. Integrated analysis of these datasets enables the identification of disease-associated, clonally expanded lymphocyte subsets, elucidation of pathogenic immune circuits, and molecular subtyping of patients. These insights inform clinical applications, including disease classification, targeted therapy selection, and prediction of treatment response. The disease annotations highlight key cell types and mediators—such as ABCs, Tph cells, Bregs, CD8^+^ T cells, and EOMES^+^ subsets—whose clonal architecture and functional properties are central to the disease mechanisms discussed in this review.

Single-cell T/B cell receptor sequencing (scTCR/BCR-seq) has revolutionized the study of immune receptors. Overcoming the averaging inherent to bulk methods, scTCR-seq precisely resolves paired α/β TCR sequences and links them to T-cell phenotypes, while scBCR-seq determines heavy/light chain pairing and reconstructs B-cell differentiation trajectories (8–10). Accurate chain pairing at single-cell resolution provides an unparalleled view of lymphocytes function and fate decisions (9, 11–13). As a result, multi-omics frameworks that integrate scTCR/BCR-seq with scRNA-seq are enabling a continuum from discovery to clinical translation and advancing autoimmune research toward precision medicine (**Figure 1**).

Despite these advances, treating autoimmune diseases remains challenging because pathogenic responses exhibit profound inter-individual heterogeneity and clonal complexity (14–16). Consequently, therapies that broadly target T or B cells often show variable efficacy, as they fail to precisely identify and eliminate the key pathogenic clones (17–19). scTCR/BCR-seq addresses this gap by correlating clonality with functional state at single-cell resolution, thereby dissecting autoimmune pathogenesis and revealing new therapeutic targets. This review summarizes the principles and development of scTCR/BCR-seq, highlights its applications in mechanistic research and targeted therapy for autoimmune diseases, and discusses its translational potential.

## 2 Single-cell TCR/BCR diversity and core advantages

### 2.1 Generation of V(D)J diversity

The diversity of T-cell and B-cell receptors underpins adaptive immunity. It arises primarily from V(D)J recombination via three mechanisms. First, combinatorial diversity is generated by the stochastic assembly of multiple gene segments (e.g., TCR: 50–70 Vα and 60–70 Jα segments; 40–50 Vβ, 2 Dβ, and 13 Jβ segments (20–22); BCR: 40–50 VH, 23 DH, and 6 JH segments for the heavy chain; 35–40 Vκ and 5 Jκ or 30–35 Vλ and 4–5 Jλ segments for the light chain (23–25)). Second, junctional diversity is introduced through the random insertion and deletion of nucleotides, a process mediated by terminal deoxynucleotidyl transferase (TdT)- at the junctions of rearranged segments. This mechanism is particularly active within the Dβ region of the TCR β chain and the DH region of the BCR heavy chain. Finally, somatic hypermutation (SHM), which is unique to BCRs, further diversifies the repertoire during affinity maturation (26). The hypervariable loops generated by these combined diversification processes are known as complementarity-determining regions (CDRs). Among them, CDR3 is the most variable in both sequence and length, as it spans the junction of the V, (D), and J segments. Positioned at the center of the antigen-binding site and making direct contact with the peptide antigen, CDR3 largely determines the fine specificity of a TCR or BCR (27, 28). Given its extreme sequence diversity, the CDR3 sequence therefore serves as a practical clonal identifier for each T or B cell clone. The vast potential repertoire generated by these mechanisms enables the abnormal selection, clonal expansion, and pathological activity of autoreactive clones upon the breakdown of immune tolerance, thereby establishing a direct molecular basis for development of autoimmune pathology (29–31).

### 2.2 Methodologies for TCR/BCR sequencing

The primary goal of TCR/BCR sequencing is the accurate capture of V(D)J-rearranged sequences, particularly the antigen-binding CDR3. Standard procedures include nucleic acid extraction, library construction, high-throughput sequencing, and computational analysis. Although the choice of starting material and the general approach to library preparation are similar for TCR and BCR analysis, primer design must be specifically adapted to the distinct genomic architecture of each receptor locus.

The selection between genomic DNA (gDNA) and RNA depends on the research question. Because it is stable and free from transcriptional bias, gDNA is well suited for inferring recombination patterns and tracing clonal phylogeny. RNA profiles the expressed repertoire, enabling direct linkage to cellular states and facilitating the identification of antigen-specific clones.

Two main strategies are used for library construction. In multiplex PCR (mPCR), overlapping V- and J-targeted primer sets amplify rearranged products. Potential amplification bias in mPCR can be reduced by optimizing primer concentrations and by incorporating unique molecular identifiers (UMIs) for digital error correction. Alternatively, 5′ RACE (rapid amplification of cDNA ends)-coupled nested PCR exploits the poly(A) tail to capture full-length variable region transcripts, preserving complete V regions and CDR3 sequences with high fidelity. This approach is particularly advantageous for precise receptor reconstruction, such as TCR-T cell screening and neutralizing antibody discovery and is often the preferred strategy for single-cell RNA-based scTCR/BCR-seq libraries.

### 2.3 Unique contributions of single-cell resolution

Bulk sequencing treats T and B cells as homogeneous populations and therefore captures only isolated chains or population-averaged characteristics. This limitation consequently obscures the relationships among receptor sequence, cell identity, and functional state. scTCR/BCR-seq overcomes these limitations through single-cell resolution and paired-chain recovery, providing two transformative advances. First, it enables the precise pairing of TCR α/β chains and BCR heavy/light chains within individual cells, which allows for definitive clonal identification. This specific insight, uniquely dependent on paired-chain information, is critical for accurately defining a clone. Second, when integrated with single-cell transcriptomics and other phenotyping modalities, scTCR/BCR−seq supports the construction of multidimensional maps that simultaneously capture receptor sequences and global transcriptional profiles. These integrative analyses resolve the clonal architecture of functionally distinct lymphocyte subsets and directly link clonal identity to functional state and differentiation trajectories.

### 2.4 Technical platforms, computational pipelines, and analytical considerations

The successful translation of scTCR/BCR−seq from a conceptual advance into a broadly applicable experimental tool depends on three key factors: the choice of single-cell platform, the computational strategies used to define clonotypes, and the analytical frameworks applied to extract biological insight. Currently, scTCR/BCR−seq is dominated by two mainstream platforms: the droplet-based 10x Genomics Chromium and the microwell-based BD Rhapsody. The 10x Genomics workflow uses 5′−end capture of transcripts coupled with V(D)J−specific enrichment primers, enabling efficient, high-throughput recovery of paired TCR α/β or BCR heavy/light chains (typically 1,000–10,000 cells per run) (32, 33). It is the most widely used platform in the field and is supported by a comprehensive software ecosystem and an extensive literature base. However, its reliance on 5′ tag−based counting restricts coverage to the terminal portion of transcripts, which can compromise assembly of full-length variable regions and limit detection of somatic hypermutation (SHM) patterns distal to the 5′ end (34, 35). BD Rhapsody employs microwell-based capture with circular amplification and offers the key advantage of full-length V(D)J sequencing, enabling comprehensive SHM analysis and detailed tracking of clonal evolution (36). It is also more tolerant of samples with modest viability (≥50%), making it particularly suitable for clinical specimens. Its main limitation is lower throughput compared with 10x Genomics.

In addition, plate-based methods such as SMART−seq and SMART−seq2 use template-switching reverse transcription to generate full-length cDNA, providing superior coverage across the entire V(D)J region and the highest gene detection sensitivity (37–39). These features make SMART−seq particularly advantageous for applications that require precise receptor reconstruction, including recombinant antibody expression and TCR−T cell engineering. Nonetheless, its relatively low throughput (96–384 cells per plate) and high per-cell cost constrain its use for large-scale repertoire profiling.

More recently, several alternative platforms—such as MobiNova, Parse Biosciences Evercode, and Singleron—have emerged to provide cost-effective and/or high-throughput options. MobiNova, a droplet-based 5′ platform, offers a cost-effective alternative to 10x Genomics, with high cell capture rates (>70%) and low doublet rates (<5%) (40). Parse Biosciences Evercode uses combinatorial barcoding and does not require microfluidic instrumentation, enabling processing of up to one million cells and providing substantial cost advantages for large-scale studies (41). Singleron is a microwell-based platform that uses circular amplification to achieve full-length V(D)J sequencing and offers flexibility for non-standard species (42).

Emerging third-generation long-read sequencing technologies further alleviate the limitations of short-read platforms. By combining long-read sequencing (e.g., PacBio or Oxford Nanopore) with existing single-cell workflows such as 10x Genomics, platforms like RAGE−seq (Repertoire and Gene Expression by Sequencing) can generate accurate, full-length TCR/BCR sequences at nucleotide resolution (43, 44). This enables comprehensive SHM analysis across the entire variable region, resolves alternatively spliced BCR transcripts, and directly informs clonal lineage reconstruction. However, per-read error rates and costs remain higher than those of short-read–only methods, although circular consensus sequencing (CCS) on PacBio and improved Nanopore base-calling algorithms are steadily narrowing this gap (44, 45).

A critical performance parameter for all platforms is chain-pairing accuracy. Although single-cell resolution inherently preserves α/β or heavy/light chain pairing, technical dropouts—where one chain fails to be reverse-transcribed or amplified—can result in incomplete receptor recovery in a subset of cells (46). In addition, doublets (two cells captured in a single droplet or well) can generate artifactual chain pairings and must be computationally identified and removed; typical doublet rates vary by platform and by cell loading concentration (47, 48). These platform-specific trade-offs, along with other technical parameters relevant to TCR/BCR sequencing, are summarized in **Table 1**.

**Table 1:** Comparison of major single-cell platforms for TCR/BCR sequencing.

Platform	Core technology	TCR/BCR support	V(D)J coverage	Chain pairing	SHM detection	Throughput & cost	Key advantages	Key limitations	References
10x Genomics Chromium (5′)	Droplet microfluidics, 5′ polyA capture	Yes	5′ end (V(D)J-enriched)	~60–75% paired	Limited (5′ proximity)	High (1,000–10,000 cells/run); moderate cost	Most widely used; excellent software ecosystem; vast literature support	Incomplete full-length V(D)J; requires viable fresh/frozen samples (>80% viability); moderate doublet rate	(32, 33)
BD Rhapsody	Microwell-based, 3′ circular amplification	Yes	Full-length V(D)J	High	Comprehensive	Medium-high throughput; moderate cost	Full-length V(D)J for SHM/clonal evolution analysis; tolerates low-viability samples (>50%); supports multi-omics	Lower throughput upper limit than 10x; smaller literature base	(36)
SMART-seq/SMART-seq2	Plate-based, template-switching	Yes	Full-length cDNA	Very high	Comprehensive	Low (96–384 cells/plate); high per-cell cost	Highest sensitivity; complete V(D)J coverage; ideal for receptor reconstruction and antibody/TCR engineering	Very low throughput; labor-intensive; highest cost per cell	(37, 38)
MobiNova	Droplet microfluidics, 5′ polyA capture	Yes	5′ end (V(D)J-enriched)	~70% paired	Limited (5′ proximity)	High; cost-effective	High cell capture rate (>70%); low doublet rate (<5%); good alternative to 10x	Fewer publications to date; smaller user community	(40)
Parse Biosciences Evercode	Combinatorial barcoding	Yes	Specific region capture	Moderate–High	Limited	Ultra-high (up to 1M cells); cost-effective	No expensive instrument required; massive cell numbers; scalable for large cohorts	Newer platform with less validation; no full-length V(D)J	(41)
Singleron	Microwell-based, circular amplification	Yes	Full-length V(D)J	High	Comprehensive	Medium-high; moderate cost	Full-length V(D)J; flexible for non-standard species (e.g., rabbit)	Smaller literature base compared with 10x and BD	(42)
RAGE-seq/10x + PacBio/Nanopore	Droplet microfluidics + long-read sequencing	Yes	Full-length (long-read)	High (10x barcodes)	Comprehensive (full V(D)J)	Lower throughput; high cost	Complete V(D)J sequence at nucleotide resolution; resolves SHM, alternative splicing, clonal evolution	Higher per-read error rate (correctable by CCS); lower throughput than short-read-only	(43, 44)

Beyond platform selection, the computational strategies used to define clonotypes and integrate them with transcriptomic data are equally important for extracting biological meaning from scTCR/BCR−seq experiments. After sequencing, raw reads are processed through specialized bioinformatics pipelines. Tools such as Cell Ranger (10x Genomics), MiXCR, and TRUST4 align V(D)J reads to germline reference databases (e.g., IMGT) and assemble paired receptor chains (49–51). A clone (or clonotype) is conventionally defined as a group of cells sharing identical V and J gene segments and identical CDR3 nucleotide or amino acid sequences (52). n B cell analyses, clustering at the amino acid level with an identity threshold of ~85–90% is often used to group clonally related cells that have undergone SHM (53). Clonal lineages and families can be further inferred using tools such as the Immcantation suite (Change−O, SHazaM, Alakazam) or Partis, which reconstruct phylogenetic trees based on shared V−gene mutations (54, 55). For integrated analysis of clonotypes and transcriptomes, computational frameworks such as Scirpy (Python) and Platypus (R) support visualization of clonal expansion on UMAP embeddings and statistical testing of clonal abundance across conditions (56, 57). The Seurat v4 workflow similarly enables joint analysis of scRNA−seq and scTCR/BCR−seq data through its weighted nearest neighbor (WNN) framework (58).

Several analytical nuances are critical for robust biological interpretation. First, distinguishing genuinely pathogenic clones from those that expand as bystanders in an inflammatory milieu remains a fundamental challenge. Clonal expansion alone does not prove pathogenicity; orthogonal evidence—such as antigen-specificity assays, correlation with clinical parameters, or functional readouts—is required to establish a causal role (59, 60). Second, clonal diversity estimates are highly sensitive to sequencing depth and cell sampling. Under-sampling of low-frequency clones can exaggerate apparent clonal dominance and underestimate diversity. Computational subsampling (rarefaction) to a uniform cell or read depth is therefore essential before comparing repertoire metrics across samples or conditions (61, 62). Third, in longitudinal studies that track clonal dynamics during disease progression or treatment, quantitative frameworks such as clone-tracking indices or Bayesian dynamical models are increasingly used to distinguish stochastic fluctuations from treatment-driven clonal contraction or expansion (59). Finally, batch effects introduced by differences in sample processing, library preparation, or sequencing runs must be addressed in multi-cohort studies using computational harmonization methods analogous to those applied in scRNA−seq analysis (63, 64).

## 3 Applications of single-cell immune receptor sequencing in autoimmune diseases

scTCR/BCR-seq has become an indispensable tool for dissecting autoimmune pathogenesis and advancing clinical translation. By leveraging its core capabilities—single-cell resolution, precise paired-chain sequencing, and seamless multi-omics integration—this technology enables high-resolution tracing of clonal lineages, reconstruction of evolutionary trajectories, and functional annotation of autoreactive T and B cells. These applications are proving indispensable for elucidating disease mechanisms, identifying candidate biomarkers, and informing the development of targeted therapies. As methodological refinements continue to expand its utility, scTCR/BCR-seq is poised to provide a critical foundation for achieving precision diagnosis and treatment in autoimmune disorders.

### 3.1 Systemic lupus erythematosus

SLE represents a systemic breakdown of immune tolerance, a process primarily driven by the hyperactivation of the type I interferon (IFN-I) signaling pathway, which culminates in multi-organ damage (65, 66). The application of scTCR/BCR-seq has substantially advanced our understanding of SLE pathogenesis by elucidating the clonal dynamics that underlie the loss of tolerance, aberrant activation, and tissue infiltration of T and B lymphocytes.

The pathogenic cascade in SLE frequently originates from a defect in B cell central tolerance. Specifically, scBCR-seq analyses indicate that autoreactive B cell clones, characterized by CDR3 motifs specific for nuclear antigens such as nucleosomes and dsDNA, evade negative selection in the bone marrow and migrate to the periphery (67). In the periphery, elevated BAFF levels and persistent IFN-I signaling promote the upregulation of survival molecules like *BCL-2* and BAFF-R in these autoreactive B cells, thereby conferring a significant survival and activation advantage. Within this dysregulated landscape, a key pathogenic subset, age-associated B cells (ABCs; CD11c^+^CD11b^+^), emerges as a central regulatory hub. Integrated scRNA-seq and scBCR-seq profiling reveals that peripheral ABCs in SLE patients not only exhibit a distinct transcriptional signature characterized by high expression of STAT1 signaling components and interferon-stimulated genes (ISGs), but also display pronounced clonal expansion and carry BCRs with specific CDR3 features, as determined by scBCR-seq. This combined molecular profile underpins a unique activation, differentiation, and tissue-homing program that facilitates their escape from peripheral tolerance checkpoints (68). Functionally, enhanced oxidative phosphorylation in these cells sustains their capacity for pro-inflammatory cytokine production, positioning them as a major reservoir of autoantibody-secreting cells.

Progression to end-organ damage is characterized by tissue-specific clonal selection and homing. Significant BCR clonal sharing between peripheral blood and kidney-infiltrating plasma cells has been observed in patients with active lupus nephritis (69). Notably, the BCRs of these kidney-resident plasma cells often exhibit specific post-translational modifications (e.g., glycosylation) within their CDR3 regions. These modifications may enhance the autoantibody affinity for the glomerular basement membrane and promote tissue penetration, thereby directly contributing to injury. Furthermore, longitudinal tracking via scBCR-seq indicates that these pathogenic plasma cell clones typically carry a significantly lower SHM burden compared to plasma cells from healthy donors, suggesting failure of germinal center negative selection and enabling poorly edited, autoreactive B cell clones to bypass normal affinity maturation and differentiate directly into tissue-destructive effectors (67).

Dysregulated T cell help and cytotoxicity are crucial to the amplification and execution of the autoimmune response in SLE. scTCR-seq reveals significant disturbances in both CD4^+^ and CD8^+^ T cell compartments. In CD4^+^ T cells, follicular helper T cells (Tfh) exhibit abnormal increases in both number and function, driving the activation, proliferation, and autoantibody secretion of self-reactive B cells. In contrast, although regulatory T cells (Treg) increase in number, their suppressive function is impaired, which compromises their ability to effectively curb the imbalance between Tfh and auto-reactive B cell activation (70). Within the CD8^+^ T cell pool, effector memory T cells (Tem) show marked oligoclonal expansion and reduced TCR diversity. These cells exhibit high express cytotoxic molecules such as granzyme K (GZMK), and the extent of their expansion correlates positively with lupus nephritis activity, underscoring their potential as biomarkers for disease activity (71). A particularly pathogenic, kidney-infiltrating PD-1^+^CD8^+^ T cell subset has been identified. Despite expressing PD-1, a marker often associated with T cell exhaustion, this subset exhibits a highly activated CD29^+^ phenotype. It contributes to renal inflammation by releasing IFN-γ and cytotoxic mediators, thereby damaging tubular epithelial cells. Preclinical studies indicate that modulating the PD-1/PD-L1 axis can alter the function of this subset and ameliorate renal injury, revealing a novel therapeutic target (72).

**Figure 2:**
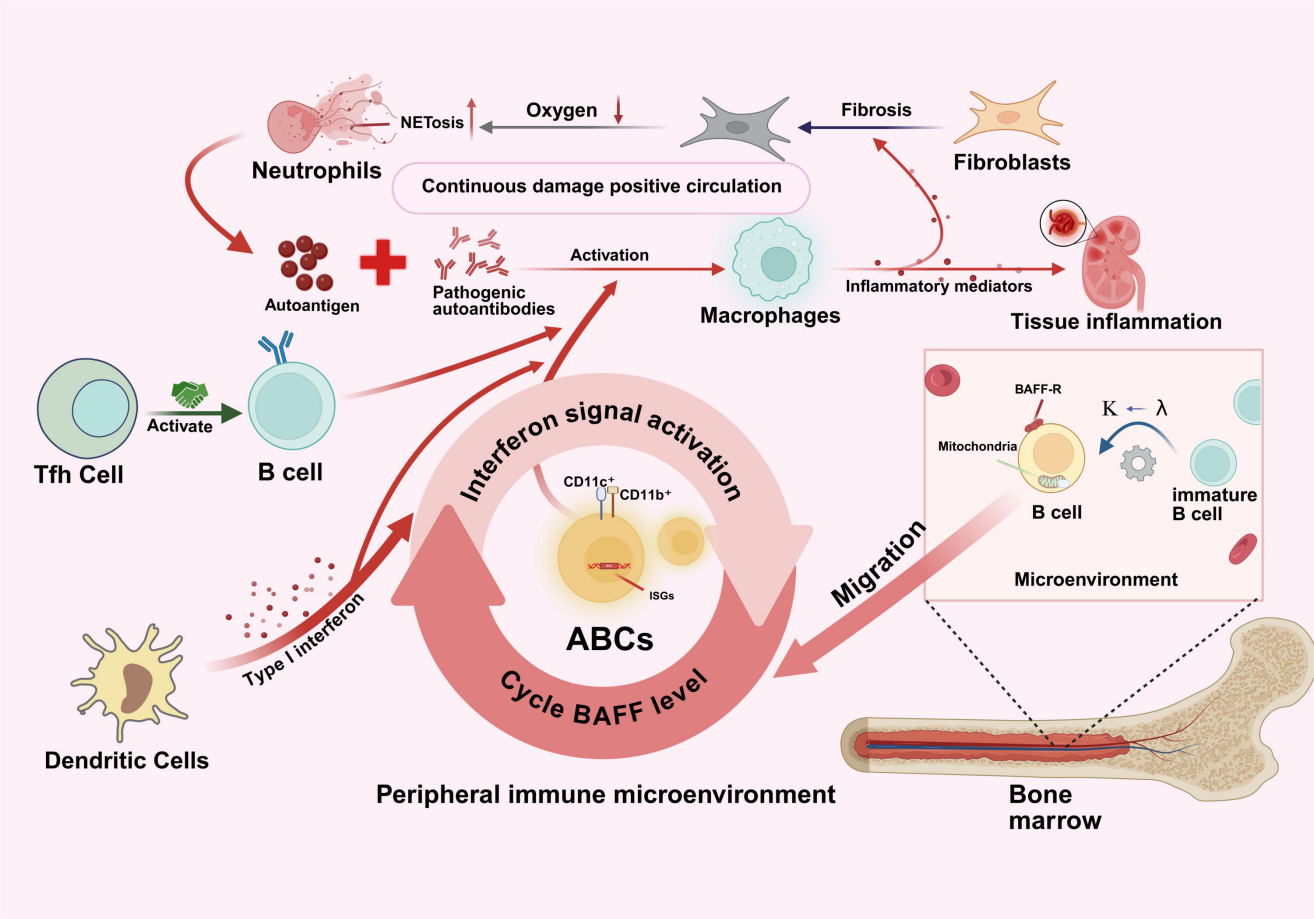
The vicious cycle of systemic immune dysregulation in SLE. This figure illustrates the pathogenic loop underlying SLE. Autoreactive B cells leave the bone marrow and enter the periphery, where type I interferon signaling activates CD11c^+^CD11b^+^ ABCs. scBCR-seq analyses demonstrate marked clonal expansion of these ABCs and reveal BCRs with characteristic CDR3 features, while scTCR-seq identifies oligoclonal expansion of cytotoxic CD8^+^ Tem cells. The autoantibodies they generate trigger neutrophil NETosis, releasing autoantigens that dendritic cells process and present. This, in turn, amplifies type I interferon signaling and immune activation, culminating in a cytokine storm and multi-organ damage.

By providing clonal-level resolution, scTCR/BCR-seq together with scRNA-seq delineate a feed-forward circuit in which innate and adaptive immunity mutually amplify each other, leading to the formation of an inflammatory storm (**Figure 2**). Specifically, scBCR-seq identifies clonally expanded ABCs as the source of autoantibody-producing B-cell clones, whereas scTCR-seq reveals the oligoclonal expansion of cytotoxic CD8^+^ Tem cells. Within this circuit, neutrophil extracellular trap (NET) formation releases waves of autoantigens, which in turn activate dendritic cells (DCs) to produce IFN-I. IFN-I then acts as a potent stimulus for ABCs and cytotoxic CD8^+^ Tem cells, driving further autoantibody and inflammatory cytokine production. The resulting immune complexes and tissue hypoxia create a feed-forward loop that further potentiates NETosis, thereby establishing a pathogenic positive feedback loop that sustains chronic inflammation and fibrosis (73).

Single-cell-based molecular subtyping is beginning to partition SLE into mechanistically distinct subsets. Notably, a subtype enriched for CD8^+^ Tem cells and ABCs, coupled with a strong IFN-I transcriptional signature, exhibits the highest disease activity (71), providing a rationale for precision and stratified therapy. scTCR/BCR-seq studies have also revealed ethnically associated heterogeneity in immune characteristics. For example, a comparative analysis of Han and Zang (Tibetan) SLE patients found that TCR abundance was notably higher in Zang patients than in controls, whereas BCR abundance was elevated in both ethnic groups; at the cellular level, CD8^+^ CTL MAL cells were the major differentially abundant subset in Han patients, while Naive CD4^+^ T cells and Memory B cells distinguished Zang patients from controls (74). These findings underscore the importance of incorporating population genetics into biomarker development and clinical trials design.

These mechanistic insights are directly informing the development of novel precision therapeutic strategies. Longitudinal scBCR-seq monitoring has elucidated the dual mechanism of belimumab, an anti-BAFF antibody, which involves the phased elimination of BAFF-R^+^ naïve B cells and the induction of apoptosis in residual pathogenic clones (75). Novel agents targeting key pathways identified through single-cell analyses, such as JAK inhibitors to block IFN-I signaling and PD-1/PD-L1 pathway modulators to target kidney-infiltrating T cells, are under active investigation (71, 72). Exploratory approaches, including combined IL-7/BAFF blockade aimed at correcting developmental tolerance defects in bone marrow precursors (e.g., the erythroblast-like EBlo subtype), offer promise for intervening at the disease source (73). Looking ahead, integrating dynamic clonal monitoring via scTCR/BCR-seq with refined molecular subtyping could enable truly personalized care by combining continuous immune surveillance, precise patient stratification, and targeted intervention to improve outcomes in SLE.

### 3.2 Rheumatoid arthritis

RA is a systemic autoimmune disease characterized by chronic, symmetrical inflammation of the synovial membrane, leading to progressive cartilage and bone destruction. The core pathology resides in a self-perpetuating disruption of the local joint immune microenvironment. This pathogenic process involves the abnormal activation of B cells and subsequent production of autoantibodies such as rheumatoid factor, anti-citrullinated protein antibodies (ACPAs), coupled with T cell-mediated inflammatory responses, and tissue damage mediated by innate immune cells. These elements intertwine to form a vicious cycle that ultimately results in joint structural damage and functional impairment (76–78). The combined application of scRNA-seq and scTCR/BCR-seq provides an unprecedented, high-resolution view into the cellular composition, clonal dynamics, and interactive networks within this microenvironment. This technological advancement is fundamentally transforming our understanding of RA pathogenesis, heterogeneity, and potential avenues for precision intervention.

Studies employing scBCR-seq have confirmed the presence of antigen-driven B cell clonal expansion within RA joints (79). Notably, the BCR clonal sharing rate is significantly higher between different affected joints, and even between the synovial tissue and synovial fluid of the same joint, than between these compartments and peripheral blood. This pattern strongly implicates a common autoantigenic driver, such as citrullinated proteins, within the joint compartment. Thus, scBCR-seq provides molecular insight into how systemic autoimmunity focuses its attack on the synovium (79).

Integrated scRNA-seq analysis has identified a key pathogenic subset: double-negative 2 B cells (DN2 B cells; IgD⁻CD27⁻CD11c^+^) (80). This subset is expanded in the peripheral blood of RA patients and is specifically enriched within inflamed synovium. Differentiation trajectory analysis positions DN2 B cells at a critical juncture on the route to becoming antibody-secreting cells (ASCs). Functionally, these cells highly express pro-inflammatory genes (e.g., DUSP4), antigen-presenting molecules (CD11c), and the transcription factor SOX5. This phenotype suggests that DN2 B cells play a dual pathogenic role: they can differentiate into ASCs to produce pathogenic autoantibodies, and they can efficiently present self-antigens to activate CD4^+^ T cells, thereby establishing a destructive B-T cell positive feedback loop that perpetuates joint inflammation Beyond mechanistic discovery, scBCR-seq-based monitoring provides critical insights into treatment response and relapse. For example, while anti-CD20 therapy (e.g., rituximab) effectively depletes most B cells, residual subsets like class-switched memory B cells with low CD20 expression, may persist. In a compensatory microenvironment with elevated factors like BAFF, these residual clones can serve as a reservoir for disease recurrence. This observation underscores the potential need for therapies that more precisely target pathogenic subsets, such as DN2 B cells (81, 82).

**Figure 3:**
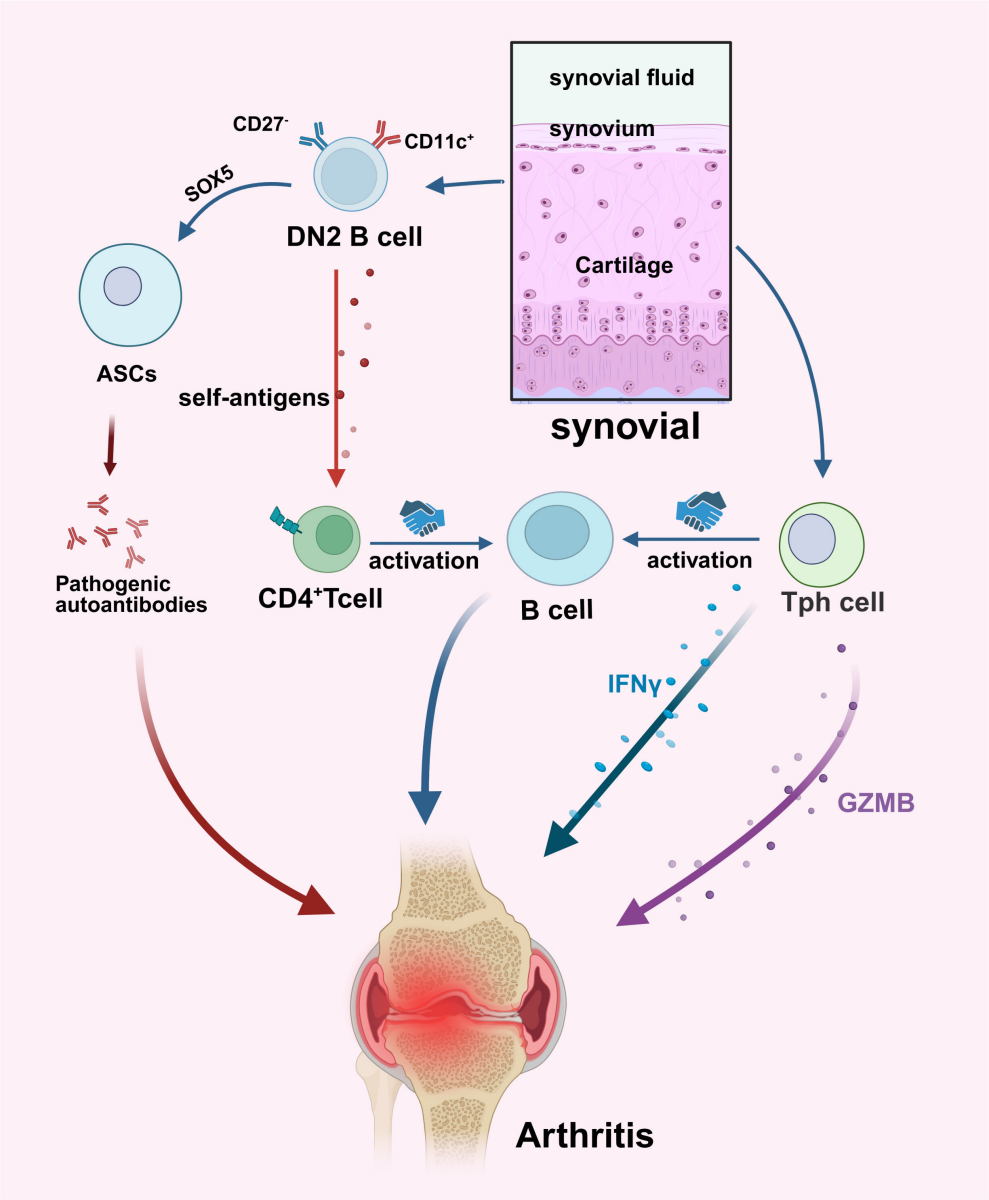
Pathogenic immune circuit mediated by DN2 B cells in RA. This figure illustrates a pathogenic immune circuit in RA mediated by DN2 B cells (IgD⁻CD27⁻CD11c^+^). scBCR-seq confirms the clonal expansion of DN2 B cells within the synovial compartment, while scTCR-seq reveals oligoclonal expansion of peripheral helper T (Tph) cells specific for citrullinated antigens. DN2 B cells differentiate into ASCs that produce pathogenic autoantibodies and, in parallel, present self-antigens to activate CD4^+^ T cells. Activated T cells release pro-inflammatory mediators, including IFN-γ and GZMB, which feedback to further activate B cells. This feed-forward loop amplifies inflammation and accelerates synovial tissue damage.

Aberrant T cell activation and differentiation constitute another core driver of RA pathology, with scTCR-seq revealing the distinct contributions of specific functional subsets. Within the CD4^+^ T cell compartment, peripheral helper T cells (Tph; PD-1^hi^HLA-DR^hi^) emerge as key instigators of local inflammation. This subset demonstrates prominent oligoclonal expansion in the synovium, and its TCRs are capable of recognizing citrullinated antigens. Tph cells directly promote tissue damage via secretion of effector molecules like IFN-γ and GZMB, while simultaneously providing help to B cells (83). Another notable CD4^+^ subset is the CD4^+^CD8α^low^ T cell, which exhibits a form of cross-reactivity by upregulating CD8α to recognize self-antigens presented by MHC class I molecules, thus bypassing traditional CD4^+^ T cell restriction. Its degree of clonal expansion correlates with disease activity, and it secretes copious amounts of pro-inflammatory cytokines, including IFN-γ, IL-17, and TNF-α, which directly fuel synovitis. The efficacy of CTLA-4-Ig (abatacept), which suppresses both the expansion and cytokine production of this subset, in correlation with clinical improvement, provides functional validation of its pathogenic role (84). In summary, these findings depict a self-amplifying immunologic circuit within the RA joint, centered on DN2 B cells. DN2 cells present antigen to activate CD4^+^ T cells, which in turn secrete cytokines that further drive B cell differentiation and autoantibody production, collectively exacerbating inflammation and tissue destruction (**Figure 3**). This circuit conceptually parallels the ABCs-centered feed-forward loop described in SLE, although RA is distinguished by its highly compartmentalized synovial microenvironment and the prominent contribution of DN2 B cells as both antibody producers and antigen presenters.

The role of CD8^+^ T cells display disease-subtype dependency. In ACPA^+^ RA, cytotoxic CD8^+^ T cells expressing GZMB can recognize citrullinated antigens (e.g., vimentin) and contribute to tissue damage by lysing synovial cells (85). However, a subset of ACPA⁻ erosive (CND-RA) disease appears to be driven by oligonally expanded CD8^+^ T cells with reduced TCRβ diversity. These clones are often enriched for cytomegalovirus (CMV)-reactivity signatures and may promote bone destruction through molecules like TNFSF14 (LIGHT), potentially independent of the classic ACPA pathway (86).. Furthermore, the characteristics of tissue-resident memory T cells (TRM) differ between inflammatory arthritis and health joint. While psoriatic arthritis synovium is rich in IL-17–producing TRM, RA synovium harbors TRM biased toward cytotoxic molecule (e.g., granzyme, perforin) expression (87). This distinction informs the rational selection of biologic therapies, for example, a preference for IL-17 inhibitors in one context versus broader T-cell-targeting agents in another.

Collectively, insights from scBCR/TCR-seq are catalyzing a shift in RA management, moving it from broad immunosuppression toward precision targeting. This shift operates on two main fronts: first, these technologies enable immune-stratified patient classification. For instance, ACPA^+^ RA (often with TRBV20–1 gene bias) and ACPA⁻ CND-RA (defined by CD8^+^ T cell oligoclonality) represent distinct molecular endotypes (88). This stratification provides a rationale for investigating targeted therapies: abatacept may benefit patients enriched for CD4^+^CD8α^low^ T cells; JAK-STAT inhibitors could mitigate cytotoxic CD8^+^ T cell signals in CND-RA; and anti-CD11c antibodies offer a strategy for selective depletion of pathogenic DN2 B cell (80, 84, 86). Second, dynamic monitoring via minimally invasive synovial biopsy coupled with single-cell sequencing allows assessment of target engagement and the early detection of resistant or recurrent clones such as tracking the evolution of residual memory B cells post-anti-CD20 therapy or monitoring pathogenic T cell clone frequencies. Such approaches enable a more precise, mechanism-based, and timely evaluation of therapeutic efficacy (88).

### 3.3 Inflammatory bowel disease

IBD, comprising primarily Crohn’s disease (CD) and ulcerative colitis (UC), is characterized by chronic, relapsing inflammation of the intestinal mucosa and impaired barrier function. In genetically susceptible hosts, its pathogenesis involves complex interactions among gut dysbiosis, aberrant activation of the mucosal immune system, and epithelial barrier defects. In particular, dysregulated B cell responses, imbalanced T helper cell differentiation, and disrupted microbiota–immune crosstalk collectively drive disease chronicity (89). Autoantibody production has also been observed and may contribute to certain manifestations, although its pathogenic role in IBD remains less well defined than in prototypical systemic autoimmune diseases. scRNA−seq and scTCR/BCR−seq have enabled high−resolution dissection of the cellular architecture, clonal dynamics, and functional interactions within the intestinal immune microenvironment. These approaches have delineated distinct immunopathological signatures between CD and UC, providing crucial insights for disease stratification and targeted therapy.

scBCR−seq studies reveal markedly different clonal architectures of B cell responses in CD versus UC. In CD, a key finding is the presence of shared B cell clones that distribute across tissues—simultaneously expanded in the inflamed intestinal mucosa, regional lymph nodes, and peripheral blood of active patients—indicating sustained, systemic antigenic stimulation. Antigen−specificity analyses show that these clones frequently utilize IGHM and IGHA isotypes and target epitopes derived from intestinal pathobionts such as Klebsiella pneumoniae and Bacillus circulants, reinforcing the role of microbial dysbiosis in CD immunopathology. Notably, although the overall SHM burden in intestinal plasma cells is lower in CD patients than in healthy controls, these cross−tissue shared clones exhibit elevated SHM. This pattern suggests repeated rounds of proliferation and mutation selection under persistent microbial drive, ultimately yielding high−affinity disease-associated clones (90). By contrast, the B cell response in UC displays pronounced mucosal compartmentalization. scBCR−seq identifies B cell clones enriched specifically in inflamed colonic regions, and these clones express high levels of the gut−homing integrin α4β7. These clones show biased usage of IGHV gene segments such as IGHV4−59 and IGHV3−48, and their CDR3 regions often contain characteristic amino acid motifs that may confer high−affinity binding to microbiota−associated antigens (90). This localized antigen drive is accompanied by altered antibody class−switching, as inflamed UC mucosa harbors an abnormally high frequency of IgG1−producing plasma cells (whereas IgA predominates in health). Through Fc−mediated engagement of Fcγ receptors on macrophages and neutrophils, these IgG1^+^ plasma cells fuel a pro−inflammatory loop that exacerbates tissue injury. scBCR−seq further confirms that these disease-associated IgG1^+^ clones are shared across B cell differentiation stages, reflecting a strong antigen−driven differentiation trajectory (91).

scTCR−seq illuminates disease−specific and spatially restricted T cell responses in IBD. In UC, the mucosa harbors an oligonally expanded population of CD8^+^EOMES^+^ TRM cells that express high levels of cytotoxic molecules and mediate direct lysis of intestinal epithelial cells. Importantly, TCR clones identical to those found in the mucosa can be detected in the peripheral blood of UC patients with extra−intestinal manifestations, suggesting these cells may migrate and contribute to systemic complications (91). In addition, immunoregulation is also impaired, although the number of Treg cells is increased in UC mucosa, aberrant up−regulation of *ZEB2* compromises their suppressive function, likely through effects on FOXP3 stability, thereby failing to restrain effector T cell activation. In CD, by contrast, T cell responses often featuring expansion of Th1/Th17−biased CD4^+^ T cell clones in chronically inflamed regions, further underscoring the immunological heterogeneity between the two IBD subtypes (92).

Collectively, single−cell analyses delineate a self−reinforcing circuit linking inflammation, microbiota, and clonal selection in active IBD. A cytokine−rich milieu (e.g., TNF−α, IL−23/IL−17) exerts selective pressure that favors the expansion of antigen−specific pathogenic clones. Oligoclonal CD8^+^ TRM cells directly damage the epithelial barrier, while locally expanded IgG1^+^ plasma cells engage FcγR−expressing innate cells to amplify neutrophilic inflammation. This process leads to a contraction of overall BCR/TCR diversity and establishes a closed loop that perpetuates mucosal immune activation and injury.

Mechanistic insights from scTCR/BCR−seq are systematically informing the shift toward precision management in IBD. First, they help explain heterogeneous responses to existing therapies. For example, scTCR−seq−based monitoring revealed that patients who fail to respond to the α4β7−integrin antagonist vedolizumab already harbor expanded clones of activated Ki67^+^ CD4^+^ memory T cells in peripheral blood before treatment; these clones preferentially use the alternative α4β1–VCAM−1 homing pathway, thereby evading drug blockade (92). This finding not only elucidates a resistance mechanism but also nominates high frequencies of α4β1^+^ activated T cell clones as a potential predictive biomarker. Second, these discoveries guide the rational design of next−generation strategies. In CD, the identification of B cell clones targeting specific pathobionts like *K. pneumoniae* supports the development of microbiota−directed interventions (e.g., precision antimicrobials or mucosal vaccines) (90). In UC, the prominence of an IgG1-driven, FcγR-dependent inflammatory axis justifies exploring FcγR-blocking approaches (91). For vedolizumab−resistant patients, dual blockade of α4β7 and α4β1 pathways emerges as a logical therapeutic alternative (92). Looking forward, the clinical utility of scTCR/BCR−seq will extend beyond baseline stratification. Its ability to dynamically track pathogenic clones during therapy will enable early assessment of treatment response and relapse risk. Further integration with spatial transcriptomics will map these clones to specific mucosal niches, ultimately guiding a paradigm shift from empirical immunosuppression toward the targeted elimination of pathogenic clones and the active remodeling of the local immune microenvironment.

### 3.4 Graves’ disease and Graves’ ophthalmopathy

**Figure 4:**
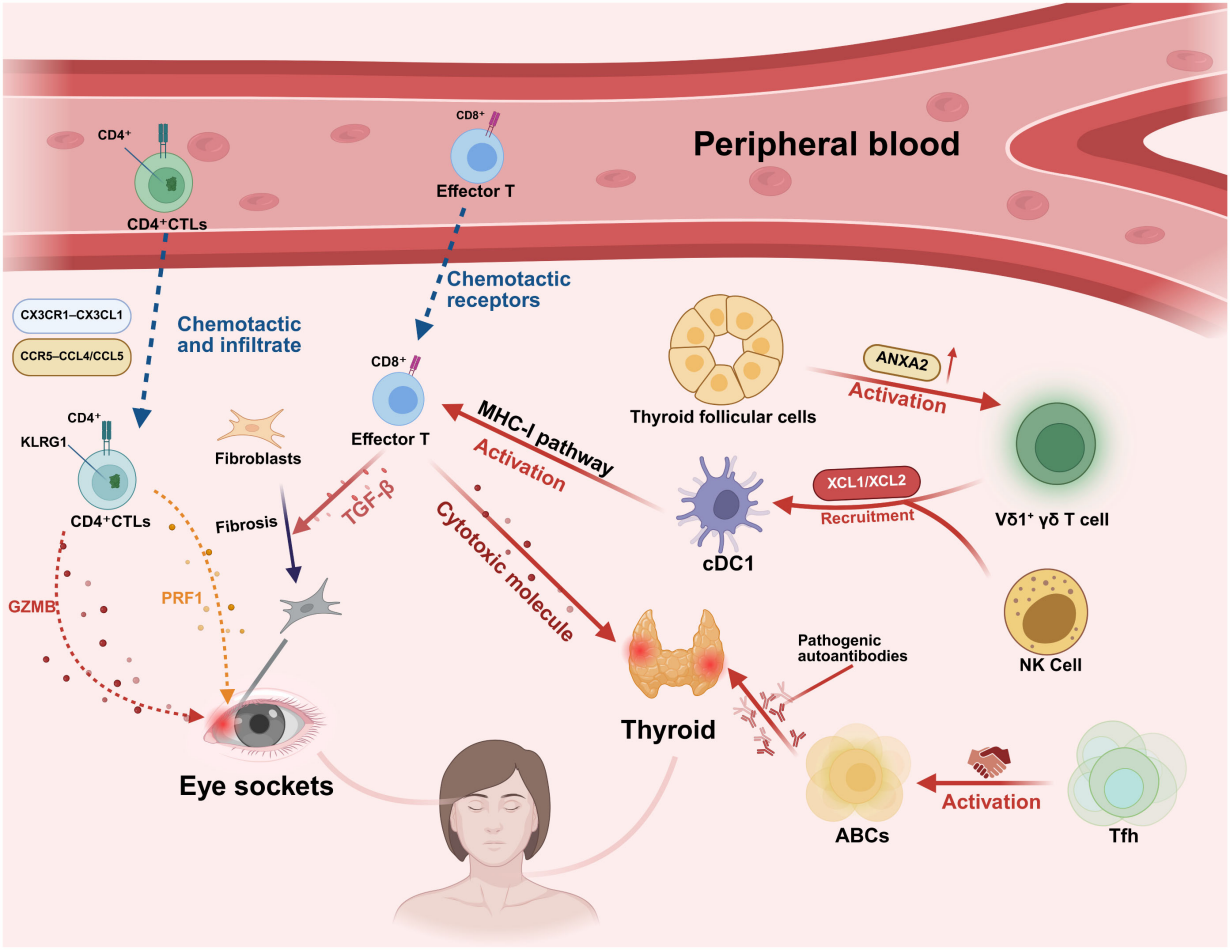
Integrated mechanism linking systemic immune activation to tissue injury in GD/GO. This diagram outlines the cascade of reactions from peripheral immune activation to tissue damage in GD/GO. scBCR-seq shows that peripheral B cell responses in GD/GO are broadly polyclonal, in contrast to the oligoclonal patterns typically observed in SLE and RA. scTCR-seq identifies TSHR-specific CD4^+^ cytotoxic T lymphocytes (CD4^+^KLRG1^+^) and CD8^+^ effector T cells undergoing clonal expansion. These autoreactive T cells and B cells (e.g., ABCs) migrate to target tissues via CX3CR1–CX3CL1 and CCR5–CCL4/CCL5 chemokine axes. In the thyroid, T cells recognize and attack follicular cells through MHC class I–restricted and ANXA2 stress-ligand–mediated pathways, engaging innate cells such as cDC1 and NK cells. In orbital tissues, infiltrating T cells release cytotoxic mediators (GZMB and PRF1) and secrete TGF-β, driving fibroblast activation and progressive fibrosis. Abbreviations: ABCs, age-related B cells; cDC1, classical type 1 dendritic cells; Tfh, follicular helper T cells.

The pathogenesis of GD and its major extrathyroidal manifestation, GO, has been classically attributed to a humoral immune response targeting the thyroid−stimulating hormone receptor (TSHR) and its autoantibodies (Thyrotropin Receptor Antibody, TRAb) (93–95). The integrated application of single-cell sequencing is revolutionizing this paradigm. Through concurrent analysis of immune cell clonality and functional states, this approach has systematically delineated the full dynamic spectrum of self-reactive lymphocytes in GD/GO—from aberrant activation to targeted tissue destruction (**Figure 4**). These insights shift the prevailing disease mechanism framework from one primarily centered on antibody-mediated effects to a novel stage emphasizing clonal evolution and functional dysregulation.

B−cell responses, profiled by integrated scRNA−seq and scBCR−seq, exhibit broad polyclonally rather than dominant oligoclonal expansion. This finding contrasts sharply with the oligoclonal B cell responses seen in diseases like SLE and RA, suggesting a different model of B cell activation in GD/GO. In active GO, peripheral regulatory B cells (Bregs) are reduced and functionally skewed toward a pro−inflammatory state (elevated *AIF1*, *CYBA*) *(*96). The overall BCR repertoire shows increased CDR3 diversity without pronounced V/J−gene bias, supporting a model of polyantigen−driven activation that may underlie chronic, multi−tissue involvement. The specific enrichment of CD11c^+^ atypical memory B cells (age-associated B cells) in localized thyroid lesions, and their robust spatial/clonal association with expanded extrafollicular Tph, directly account for the paradox of sparse germinal centers yet persistent high autoantibody titers in GD, underscoring a Tph–B cell axis-driven extrafollicular antibody response mechanism (97).

Analysis of paired scTCR-seq and scRNA-seq data maps the full dynamic spectrum of pathogenic T cell clones, including their origin, expansion, migration, and ultimate effector functions (**Figure 4**). In the local GD thyroid tissue, significant oligoclonal expansion is observed in CD8^+^ effector/memory T cells and Tph cells, which share clonality with TRM cells, revealing a process of antigen-driven local clonal selection and evolution. Moreover, a novel, MHC-II-independent pathogenic pathway was identified: under oxidative stress, thyroid follicular cells upregulate stress ligands (e.g., ANXA2) to activate tissue-resident Vδ1+ γδ T cells. This activation, in synergy with NK cells, leads to the secretion of XCL1/XCL2, which recruits cDC1 cells—completing a cascade that translates local tissue stress into a coordinated immune response (97). Peripherally, two pathogenic T−cell subsets are implicated (**Figure 4**). TSHR−specific CD4^+^ cytotoxic T lymphocytes (CTLs; CD4^+^KLRG1^+^) express high levels of GZMB, PRF1, and CX3CR1, recognize the immunodominant TSHR289 epitope, and infiltrate the orbit via CX3CR1–CX3CL1/CCR5–CCL4−CCL5 axes (98). CD8^+^ effector T cells carry genetic risk variants (e.g., SLC35G1, IDNK) and exhibit an altered clonal landscape (99). Within orbital tissue, CD4^+^ CTLs mediate acute cytotoxicity, whereas CD8^+^ T cells promote fibrosis via TGF-β. Concomitant functional conversion of local regulatory T cells (e.g., KLRC1, FGFBP2 up-regulation) further disrupts immune homeostasis (100).

These findings provide potential ways for precision medicine. Peripheral TSHR−reactive T−cell clone frequency, CD4^+^KLRG1^+^ CTL abundance, or risk−gene expression levels may serve as dynamic biomarkers for disease activity, GO progression, and treatment response. Therapeutically, strategies could include blocking pathogenic clone homing, neutralizing cytotoxic functions, modulating immune crosstalk, or exploiting clonotypic sequences for adoptive therapy or tolerance induction. Longitudinal scTCR/BCR−seq will be crucial for evaluating how existing and emerging therapies reshape the pathogenic clonal repertoire, enabling molecularly guided treatment decisions.

### 3.5 Primary Sjögren’s syndrome

PSS is a systemic autoimmune disease defined by progressive lymphocyte infiltration and destruction of exocrine glands, representing a broad collapse of immune tolerance (101). Single−cell omics approaches have systematically mapped the pathogenic continuum of pSS—from peripheral immune dysregulation to gland−targeted damage—through the lens of clonal evolution and cellular dysfunction.

The immunopathological essence of pSS originates from defects in central and peripheral immune tolerance, with B-cell abnormalities serving as the core driving factor in disease development. The B-cell receptor repertoire in PSS patients exhibits significant genetic selection bias, such as abnormally elevated usage frequencies of gene fragments like IGHV1–69 and IGHV4-30-4. This bias is clinically relevant, as *IGHV1-69* usage is strongly associated with anti-SSA antibody positivity, suggesting autoantigen-driven clonal selection and expansion. Crucially, peripheral blood memory B cells from anti-SSA/SSB double-positive patients harbor enriched VDJ transcripts lacking SHM, most notably the auto-reactive IGHV4–34 clone. The antibody encoded by this clone specifically binds erythrocyte surface antigens. Its unmutated state indicates it evaded SHM screening during peripheral tolerance, potentially representing an original auto-reactive clone that slipped through central tolerance screening, becoming a subsequent pathogenic hazard (102). Corresponding to this finding, peripheral blood from pSS patients (especially those double-positive for anti-SSA/SSB antibodies) exhibits abnormal differentiation characterized by elevated proportions of naive B cells and reduced memory B cells (102). This deviation from the normal trajectory of differentiation toward memory cells following antigen stimulation suggests potential differentiation arrest or abnormal survival. However, this peripheral differentiation arrest does not represent the functional endpoint. scBCR-seq analysis of affected target organs (e.g., labial glands) revealed that infiltrating memory B cells within the glands not only exhibited significant oligoclonal expansion but also demonstrated clonal similarity across glands from different patients (103). This demonstrates that peripheral autoreactive B cells migrate to the glandular microenvironment, where, under sustained local antigenic stimulation, they undergo clonal expansion and effector differentiation, directly contributing to local immune complex formation or amplifying T cell responses through antigen presentation. Furthermore, B cells from pSS patients consistently exhibit high activation of ISGs. Type I interferons significantly lower the activation threshold of B cells by activating signaling pathways such as STAT1, enabling low-affinity auto-reactive B cells to be more readily activated and proliferate. This establishes a vicious cycle that continuously amplifies B-cell autoimmunity (102).

T−cell abnormalities are equally critical, driving tissue injury through effector hyperactivity and impaired regulation. scTCR−seq shows reduced TCR diversity in peripheral blood, with oligoclonal expansions in Th1 and Th17 subsets carrying disease−associated CDR3 motifs (e.g., TRAV8−2/J5 | VVSDTVLETAGE), reflecting antigen−selected T−cell proliferation (104). Regulatory dysfunction is prominent: although Treg numbers may be elevated peripherally, the frequency of key functional subsets (e.g., Helios^+^ Tregs) inversely correlates with disease activity (105), and gland−infiltrating *ZEB2*^+^ Tregs display defective suppressive capacity (106). The direct effectors of gland damage are CD8^+^ TRM. Single−cell studies delineate their development: CXCR6^+^GZMK^+^ CD8^+^ T−cell precursors are recruited from blood to salivary glands via the CXCR6–CXCL16 axis; within the IL−15−rich glandular niche, they differentiate into long−lived CD69^+^ TRM. These resident cells kill glandular epithelium via granzyme/perforin and release IFN−γ and TNF−α, recruiting and activating additional immune cells (B cells, fibroblasts), thereby establishing a self−sustaining inflammatory circuit that fuels progressive tissue destruction (104–107).

Single−cell−based stratification reveals distinct therapeutic opportunities. For the interferon−driven subset, JAK inhibitors or anti−BAFF agents (e.g., belimumab) may mitigate disease by suppressing aberrant B−cell activation (102). For the cytotoxic TRM−enriched phenotype, targeting recruitment (CXCR6 inhibitors) or differentiation/survival (IL−15 pathway blockers) holds promise (106, 107). Furthermore, pathogenic B−cell clones identified by scBCR−seq (e.g., specific IGHV genotypes) and autoreactive T−cell clones defined by scTCR−seq provide precise molecular targets for adoptive cellular therapies (CAR−T, engineered Tregs) or antigen−specific tolerance strategies.

### 3.6 Multiple sclerosis

MS is an autoimmune disease characterized by the migration of peripherally activated pathogenic T and B cells across the blood-brain barrier to attack myelin and oligodendrocytes in the central nervous system (CNS), leading to demyelination and neurological impairment (108, 109). Single-cell multi−omics technologies have provided crucial insights into MS pathogenesis, disease subtyping, and precision therapy by resolving the clonal dynamics and functional heterogeneity of CNS−infiltrating immune cells (110), an analysis made uniquely powerful by access to the cerebrospinal fluid (CSF) compartment.

The CSF of MS patients display hallmarks of antigen−driven B−cell activation. Memory B cells and plasmablasts/plasma cells, predominantly IgM^+^ and IgG1^+^, are specifically enriched in the CSF. Their BCR repertoire exhibits significantly lower clonal diversity than that in peripheral blood, accompanied by a distinct bias toward IGHM and IGHG1 usage, indicative of self−antigen−driven selection (111). Functionally, CSF−resident memory B cells maintain clonal expansion by activating the NF−κB signaling and cholesterol synthesis pathways. Mature plasma cells disrupt local immune homeostasis by suppressing the SMAD/TGF−β1 anti−inflammatory pathway and secrete IL−16 to recruit CD4^+^ T cells, thereby amplifying the inflammatory cascade. Importantly, clonally expanded IgG1^+^ plasma cells secrete anti−myelin autoantibodies that directly mediate oligodendrocyte injury and myelin loss (111).

T cell responses in MS show subset specificity and vary with disease stage. CD4^+^ cytotoxic T cells, particularly a CD4^+^EOMES^+^ subset enriched in the CSF, express high levels of cytotoxic molecules such as GZMB and perforin and can directly recognize and damage neural cells. Tfh cells enhance B cell activation and antibody production by secreting key factors such as IL-21, thereby indirectly exacerbating CNS damage (112). In secondary progressive MS (SPMS), terminally differentiated effector memory T cells (TEMRA) expand abnormally in peripheral blood. This subset preferentially utilizes TRBV9/TRAV1−2 TCRs, suggesting recognition of CNS autoantigens via molecular mimicry. Their elevated GZMB expression is regulated by the transcription factor T−bet, and their peripheral frequency correlates positively with neurological disability and disease progression rate, making them a potential biomarker for distinguishing SPMS from relapsing−remitting MS (RRMS) (113). Furthermore, the CSF of MS patients is enriched for activated CD8^+^ T cells with cytotoxic and tissue−resident features. Their TCRs specifically recognize Epstein−Barr virus (EBV) antigens (e.g., EBNA3A). Through EBV−directed cross−reactivity, these cells release GZMB to kill oligodendrocytes, providing direct evidence for a central role of EBV in MS pathogenesis (114).

These mechanistic insights are shifting MS treatment from broad immunosuppression toward precision−targeted interventions. Potential strategies include depletion of CSF−resident B cells using anti−CD20 monoclonal antibodies or blockade of Tfh−B cell interactions to suppress autoantibody production (112). For pathogenic T−cell subsets, anti−VLA−4 antibodies can inhibit CNS migration of EOMES^+^ CD4^+^ T cells, while T−bet inhibitors may downregulate the cytotoxicity of CD8^+^ TEMRA cells (113). Approaches such as EBV vaccination or elimination of EBV−reactive CD8^+^ T cells aim to sever the link between viral infection and autoimmunity. By identifying the CSF as a major site of pathogenic clone expansion, deciphering EBV−driven T−cell cross−reactivity, and characterizing stage−specific biomarkers (e.g., CD8^+^ TEMRA), single−cell technologies are advancing MS into an era of precision intervention. Future integration of spatial transcriptomics with blood−brain barrier−penetrating drug delivery systems holds promise for curative strategies that combine clonal eradication with active remodeling of the CNS microenvironment.

### 3.7 Type 1 diabetes

T1D is a chronic autoimmune disease in which a breakdown of immune tolerance toward islet antigens such as insulin drives the progressive destruction of pancreatic β-cells. This pathology is mediated by autoreactive T and B lymphocytes that infiltrate the islets and disrupt β−cell function (115, 116). Single−cell technologies have not only elucidated the dynamic evolution of pathogenic clones but also provided critical support for staging, prevention, and precision intervention in T1D (117). Realizing this potential depends fundamentally on a clear understanding of stage−specific immune abnormalities.

Disease progression typically begins with early humoral dysregulation. Using scBCR−seq, islet−antigen−reactive B cells (IARBs) have been identified and characterized in peripheral blood from autoantibody−positive preclinical subjects and recently diagnosed patients. IARBs show significant oligoclonal expansion in both groups, indicative of antigen−driven selection. Notably, in the preclinical phase, polyreactive IARB clones are particularly prominent and frequently utilize IGHV genes associated with autoimmunity, such as IGHV4−34 and its close homolog IGHV4−4. These B cells may be abnormally activated through dysregulated BCR signaling (e.g., reduced LYN kinase expression) and/or cross−reactivity with exogenous pathogens such as EBV. Beyond secreting autoantibodies, activated IARBs also serve as efficient antigen−presenting cells that prime and amplify autoreactive T−cell responses, thereby playing a dual role in both humoral and cellular arms of the autoimmune attack (118).

As disease advances, T−cell−mediated immunity becomes the dominant driver of β−cell loss. CD4^+^ T cell responses evolve dynamically: during the preclinical phase, public TCR clones that recognize multiple islet antigens—often bearing germline−like TCRα chains with short CDR3 regions—can be detected, suggesting common early immune triggers (119). By clinical onset, epitope spreading leads to a more individualized, patient−dominant TCR repertoire. One key subset, BHLHE40−high pro−inflammatory memory CD4^+^ T cells, produces GM−CSF and TNF−α, and its baseline frequency correlates with resistance to CD2−targeted therapy, highlighting its potential as a predictive biomarker (120). CD8^+^ T cells, the main effectors of β−cell killing, express high levels of cytotoxic molecules (e.g., PRF1, GZMH) in human T1D and display biased TRAV17/TRAV21 usage, enabling migration into islets. This enhanced cytotoxicity coincides with impaired Treg function due to elevated IL4R and TNFRSF4 expression, tipping the balance toward destruction (121). Animal models further reveal heterogeneity in CD8^+^ T−cell phenotypes: PD−1 inhibitor−induced T1D is dominated by CD38^+^NKG2D^+^ terminally exhausted−like CD8^+^ T cells, whereas spontaneous NOD mice show a preponderance of effector/memory subsets (122). These distinctions underscore the importance of monitoring peripheral CD8^+^ T−cell clonal dynamics for clinical risk stratification.

Collectively, these stage−specific insights inform a precision, phase−adapted interventional framework for T1D. In the autoantibody−positive (AAB) phase, strategies may include targeting IGHV4−4^+^ IARBs with anti−CD20 antibodies or blocking EBV cross−reactivity. During early clinical onset, eliminating public CD4^+^ clones or inhibiting CD2 signaling could halt progression. In established disease, JAK inhibitors may help suppress TRAV17^+^ CD8^+^ T−cell cytotoxicity to preserve residual β−cell function. Treatment response can be further guided by biomarker thresholds; for example, a BHLHE40^+^ CD4^+^ T−cell frequency >15% may indicate resistance to CD2−targeted therapy, and expansion of peripheral CD38^+^ CD8^+^ T cells could signal risk for PD−1 inhibitor−induced T1D. Overall, these findings are shifting the T1D therapeutic paradigm from broad immunosuppression toward personalized, mechanism−based intervention.

### 3.8 Psoriasis

Psoriasis is a chronic autoimmune/autoinflammatory disease characterized by keratinocyte hyperproliferation and chronic inflammation, frequently associated with systemic complications such as arthritis (123). While traditional research focused on local effector cells (e.g., skin−resident Th17 cells), single−cell TCR/BCR and transcriptome sequencing have broadened the view to encompass the dynamic migration, tissue−specific differentiation, and long−term residency of disease-associated lymphocyte clones. This clonal−level view systematically explains the transition from localized skin inflammation to systemic, chronic, and joint−invasive disease (108, 124).

Understanding psoriasis pathology requires connecting systemic and local immune responses at a clonal level. Humoral immunity participates systemically: peripheral blood plasma cells, predominantly IgA1^+^/IgG1^+^, show clonal expansion, reduced BCR diversity, and biased usage of IGHV3 gene segments. Importantly, the frequency of these clones correlates positively with skin−lesion severity, confirming their role in amplifying systemic inflammation (125). T cells, however, emerge as the central drivers. Single−cell studies challenge the traditional view by identifying non−skin−homing (CLA⁻) circulating T cells as key mediators of systemic inflammation. T−cell clones infiltrating skin lesions overlap more significantly with CLA⁻ than with CLA^+^ circulating clones, and CLA⁻ clones exhibit higher expansion frequencies. By recognizing antigens shared between skin and joints and secreting TNF−α and IFN−γ, these cells establish a skin–joint immune axis that underpins chronic lesion recurrence and progression to psoriatic arthritis (PsA) (126).

Within the local skin microenvironment, single−cell sequencing reveals how autoreactive T cells initiate and sustain inflammation. A population of IL−26^+^ Th17−intermediate cells, resident in lesions as CD103^+^ TRM, recognizes local self−antigens (e.g., ADAMTSL5, LL37). Through IL−26 secretion, they stimulate keratinocytes to produce TGF−β1, which in turn promotes their differentiation into mature IL−17A−producing Th17 cells, creating a self−amplifying inflammatory circuit (127). Furthermore, lesion−enriched Th17 and Tc17 cells display marked clonal expansion that correlates with disease severity. Their TCRs carry disease−associated motifs and they highly express effector molecules (IL−17, GZMB, CXCL13), directly fueling the IL−23/IL−17−driven inflammatory axis (87).

When psoriasis progresses to PsA, T cell clones trace a path from systemic circulation to joint−specific adaptation. Joint damage involves distinct migratory and resident clones. CCR4^+^ CD8^+^ central memory T cells (TCM), already clonally expanded in the blood of psoriasis patients, further accumulate in PsA synovial fluid and differentiate into cytotoxic effectors (GZMK^+^, GZMB^+^), acting as bridging cells that link skin and joint inflammation (128). Within the synovium, a heterogeneous pool of CD8^+^ TRM forms. IL−17−secreting TRM (CD161^+^CCR6^+^) possess a diverse, independent TCR repertoire, likely recognizing unique joint antigens, and sustain chronic synovitis via IL−17A and TNF−α production. Cytotoxic TRM clones overlap with infiltrating effector T cells, suggesting a common precursor and a cooperative role in joint destruction (87). The long−term persistence and functional polarization of these pathogenic clones underpin PsA chronicity. These single−cell−derived insights are clarifying precision management strategies for psoriasis by aligning therapeutic interventions with distinct immunopathological mechanisms. For patients with skin−limited disease, where local activation of the IL−23/IL−17 axis predominates, monoclonal antibodies targeting IL−23p19 or IL−17A effectively disrupt the focal inflammatory circuit. In contrast, a systemic−inflammatory phenotype—characterized by clonal expansion of non−skin−homing (CLA⁻) T cells and activated plasma cells, often accompanied by metabolic comorbidities—may be better addressed with systemic agents such as JAK inhibitors, which can modulate global immune dysregulation and potentially attenuate the risk of progression to PsA. Once PsA is established, a dual therapeutic approach appears warranted: first, intercepting the homing of pathogenic clones to the joint, for example by targeting receptors like CCR4, and second, neutralizing effector molecules produced by already−resident pathogenic T cells, notably through IL−17 inhibition—a strategy whose pronounced efficacy in PsA likely stems from the unique enrichment of IL−17−secreting tissue−resident memory T cells within the inflamed synovium. In contrast, conventional broad−spectrum immunosuppressants (e.g., methotrexate, glucocorticoids) alleviate symptoms but fail to eradicate the TCR−driven, tissue−resident immune memory that perpetuates disease. This underscores the clinical advantage of precision targeting based on molecular and clonal subtyping. Moving forward, integrating spatial transcriptomics to map clonal niches within tissue microenvironments, along with developing novel therapies capable of durably eliminating or modulating pathogenic resident clones, will be essential to shift psoriasis treatment from symptomatic control toward restoration of immune homeostasis.

### 3.9 Pemphigus vulgaris

PV is an autoimmune bullous disease characterized by autoantibodies targeting desmogleins (Dsg), adhesion molecules critical for epidermal integrity (129, 130). The core pathogenesis involves the breakdown of cutaneous immune privilege by autoreactive T and B cells, which organize into tertiary lymphoid structures (TLS) at lesion sites. Within these TLS, sustained production of pathogenic anti-Dsg antibodies and direct cytotoxic effects synergistically drive epidermal acantholysis and blister formation (131–133).

Single-cell multi-omics studies have established the TLS microenvironment as a pathogenic hub, wherein B and T cells engage in a coordinated network. ASCs dominate the lesions, exhibiting prominent oligoclonal expansion, a high burden of somatic hypermutation, and preferential class-switching to the IgG4 isotype. The BCRs of these ASCs often contain CDR3 motifs compatible with Dsg epitopes. Sustained activation through pathways such as TLR–NF-κB enables these cells to serve as a long-lived reservoir of pathogenic antibodies (134, 135). These plasma cells may arise either from pre-committed Dsg-specific B cells or from naïve/non-specific B cells recruited to the TLS and subsequently undergoing antigen-driven differentiation locally (136). Concurrently, a population of CXCL13^+^ CD4^+^ T cells—displaying features of both tissue-resident memory and follicular helper cells—has been identified as a key orchestrator of TLS function. This subset undergoes Dsg-specific clonal expansion and perpetuates autoimmunity via a self-reinforcing circuit: dysfunctional regulatory T cells Tregs produce TGF-β, which stimulates CXCL13 secretion; CXCL13 then recruits B cells to the TLS, and autocrine IL-21 from the CXCL13^+^ CD4^+^ T cells directly drives the differentiation of recruited B cells into ASCs, thereby establishing a self-sustaining pathogenic axis (133).

Tissue injury results from a combination of unchecked effector mechanisms and impaired immunoregulation, with distinct patterns across disease subtypes. In classic PV, lesions are enriched for CD69^+^IFN-γ^+^ CD8^+^ TRM, which show higher clonality compared to peripheral blood. These cells directly disrupt keratinocyte adhesion through the release of cytotoxic granules and activation of the Fas/FasL apoptosis pathway (137). In contrast, tissue damage in bullous pemphigoid (BP) appears more dependent on infiltrating CD8^+^ effector memory T cells operating through granzyme-mediated cytotoxicity (136). Immunoregulatory deficits also differ: PV patients often have numerically normal but functionally impaired Tregs, marked by diminished FOXP3 expression and activity, whereas BP patients exhibit expanded but exhausted-like Treg populations with upregulated inhibitory receptors (136) (137). Moreover, myeloid cells such as macrophages amplify inflammation by secreting type I interferons and IL-1α, thereby recruiting additional leukocytes and synergizing with CD8^+^ TRM-mediated damage to exacerbate tissue injury (137).

Conventional therapies, including glucocorticoids or B-cell–depleting agents like rituximab (anti-CD20), provide symptomatic control but frequently fail to eradicate the chronic immune memory maintained by long-lived, TLS-resident plasma cells and TRM, particularly as differentiated CD20⁻ plasma cells are largely resistant to such treatments (135, 138). Insights from single-cell studies are guiding more precise therapeutic strategies. These include disrupting the TLS niche itself by blocking the CXCL13–CXCR5 axis or IL-21 signaling (134); targeting subtype−dominant pathways, such as using JAK inhibitors to suppress IFN-γ signals in PV or low-dose IL-2 to revive exhausted Tregs in BP; and exploring disease−rooted approaches like anti-BCMA CAR-T cell therapy to directly eliminate long-lived plasma cells, the primary source of pathogenic antibodies. Collectively, these strategies exemplify a paradigm shift from broad immunosuppression toward precision intervention based on disease−specific immune phenotypes and clonal architecture.

### 3.10 Comparative insights across autoimmune diseases

**Figure 5:**
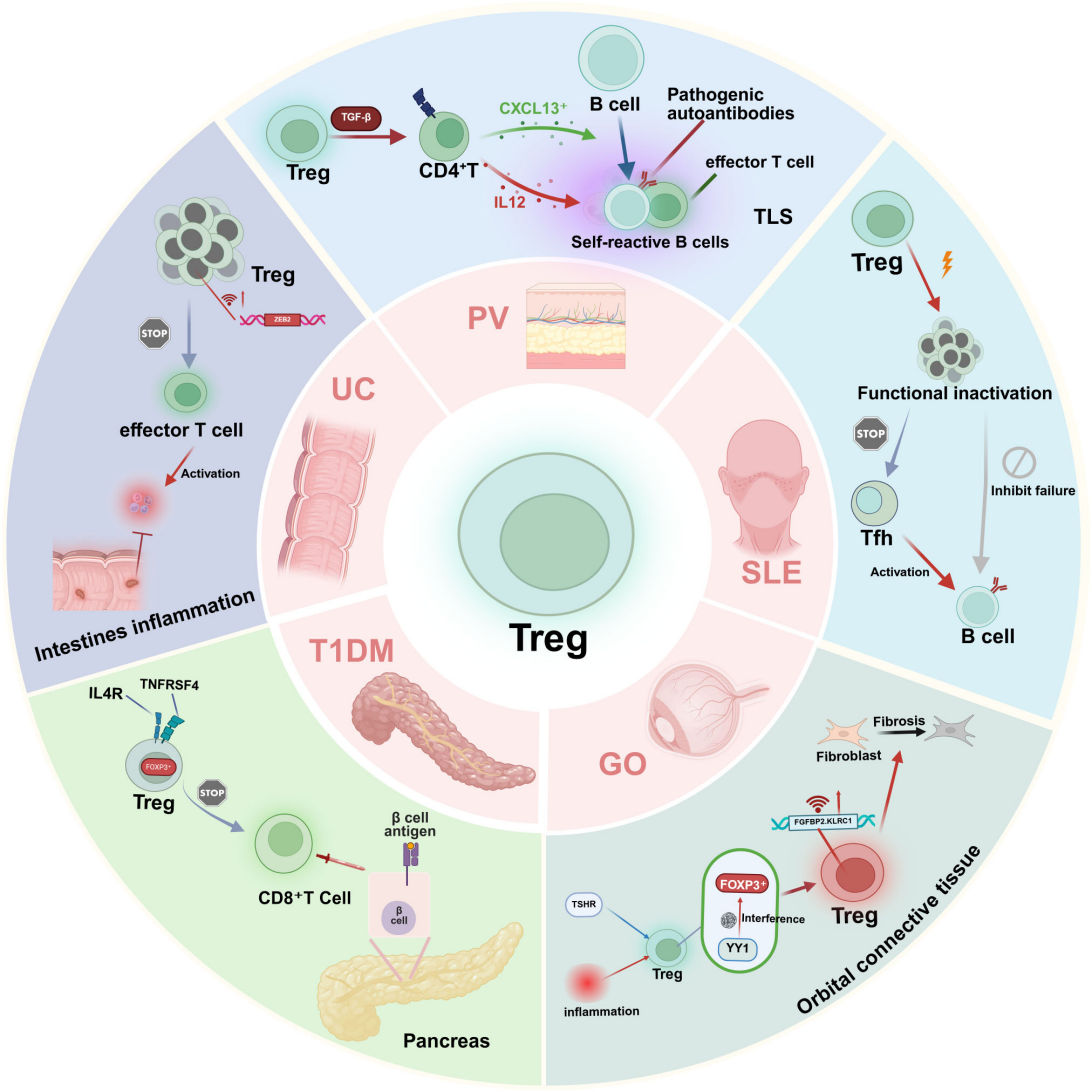
Dysfunction and heterogeneous manifestations of Tregs in different autoimmune diseases. This figure depicts disease-specific patterns of Treg dysfunction across autoimmune disorders. These distinct functional states were defined by integrating scTCR-seq with scRNA-seq, which together delineate the clonal origins and transcriptional programs of aberrant Tregs in each disease. In SLE and UC, Treg numbers may be normal or increased, yet their suppressive capacity is compromised, leaving them unable to effectively restrain effector T cells. In T1D, localized Treg dysfunction in the pancreas fails to control self-reactive CD8^+^ T cell that target β cells. In PV, Tregs within TLS undergo functional reprogramming into a Tfh-like phenotype that actively supports pathogenic B cell responses. In GD/GO, orbital Tregs contribute to fibroblast activation and collagen deposition, thereby promoting fibrosis.

A common feature across these conditions, beyond the disease-specific clonal expansion of effector cells, is the widespread dysregulation of the immune regulatory system, particularly the functional impairment of Tregs. However, this defect manifests distinctly in different diseases (**Figure 5**). For instance, in T1D, the failure is more localized, with pancreatic Tregs unable to control autoreactive CD8^+^ T cell attacks on β-cells. A more extreme alteration is observed in the tertiary lymphoid structures of pemphigus vulgaris, where Tregs can convert to a pro-inflammatory, T-follicular-helper-like phenotype that actively supports pathogenic B cell responses. In GO, Tregs contribute directly to pathology by promoting fibroblast activation and tissue fibrosis. scTCR-seq combined with transcriptomic analysis is being utilized to precisely decipher the clonal origins and functional states of these aberrant Tregs.

Beyond Treg biology, a closer comparative analysis shows that the transformative impact of scTCR/BCR−seq has not been uniform across autoimmune diseases. In diseases like SLE and MS, where the trafficking and clonal evolution of lymphocytes between peripheral blood, target organs, or immune-privileged sites define the pathology, this technology has provided profound, otherwise unattainable insights by directly linking specific clonotypes to tissue infiltration and functional states. In contrast, for a condition like GD, where B cell responses are broadly polyclonal, the primary utility of scTCR/BCR-seq may lie in identifying rare pathogenic T cell clones or characterizing the functional states of non-clonal immune populations, rather than tracking dominant B cell expansions. Similarly, in RA and psoriasis, where clonal expansion of both T and B cells contributes to disease, the technology has been particularly useful for resolving the clonal architecture of joint− and skin−infiltrating lymphocytes and for associating specific clonotypes with disease progression and treatment response.

The studies reviewed here (**Table 2**) distill a core principle: despite their differing pathological foci and clinical phenotypes, single-cell analysis shows that immune dysregulation operates along two interrelated axes. The first is a spectrum of clonal focusing. At one end are diseases characterized by highly focused, oligoclonal expansion of autoreactive lymphocytes—exemplified by SLE, RA, and PV—in which scTCR/BCR−seq has been crucial for identifying dominant pathogenic clones. At the other end are conditions marked by broad, polyclonal humoral activation, such as GD/GO, where the main contribution of this technology has been to delineate the functional states of non−clonal populations. The second axis is a spectrum of effector modalities. Cytotoxic CD8^+^ TRM cells dominate tissue destruction in UC, pSS, and PV, whereas Th17−mediated pathways are more prominent in psoriasis and PsA. What unites these conditions is a fundamental imbalance between effector and regulatory functions—an imbalance that scTCR/BCR−seq is uniquely suited to resolve at the clonal level. Clarifying this differential impact and these distinct modes of clonal dysregulation is essential for guiding future research investments, prioritizing diseases in which clonal tracking is most likely to yield clinically actionable targets and ultimately enabling precision intervention.

**Table 2:** Overview of key results from integrated scRNA-seq and scTCR/BCR-seq analyses across multiple autoimmune diseases.

Autoimmune diseases	Methods	Cell source	Main discovery	Clinical association	References
SLE	scRNA-seq combined with scTCR/BCR-seq	Peripheral blood, kidney	Defective central tolerance in B cells, reduced κ→λ receptor editing efficiency; age-related B cells serve as a key hub for peripheral activation; oligoclonal expansion of CD8^+^ effector memory T cells.	Treatment response monitoring (e.g., belimumab eliminates BAFF-R^+^ naive B cells), disease activity assessment (CD8^+^ TEM frequency correlates with nephritis severity), precise subtyping (e.g., interferon-highly activated C2 subtype), and novel therapeutic targets (e.g., JAK inhibitors, PD-1 pathway modulation).	(67, 68, 71)
RA	scRNA-seq combined with scTCR/BCR-seq	Synovial tissue, peripheral blood	Antigen-driven clonal expansion occurs within synovial tissue; DN2 B cells serve as a key pathogenic hub; oligoclonal expansion of CD4^+^CD8αlow T cells.	Guiding targeted therapies (e.g., abatacept targeting CD4^+^CD8αlow T cells, anti-CD11c targeting DN2 B cells), patient stratification (based on ACPA status and T/B cell clonal characteristics), and treatment response monitoring (monitoring residual clones after rituximab therapy).	(79, 80, 84)
IBD	scRNA-seq combined with scTCR/BCR-seq	Intestinal mucosa, lymph nodes, peripheral blood	Crohn’s disease patients exhibit BCR clones shared across tissues; ulcerative colitis features mucosal homing B-cell responses with specific CDR3 motifs.	Biomarker discovery (shared clones as CD markers), guiding precision therapy (e.g., targeting pathogenic bacteria for CD, targeting FcγR or combined integrin blockade for UC), predicting treatment response (e.g., Ki67^+^CD4^+^ T cells predicting non-response to vedolizumab).	(90–92)
GD/GO	scRNA-seq combined with scTCR/BCR-seq	Peripheral blood, orbital tissue	Reduced proportion and function of regulatory B cells; clonal expansion of CD8^+^ effector T cells and CD4^+^ cytotoxic T lymphocytes; conversion of Tregs to a cytotoxic phenotype.	Uncovering the pathogenesis and guiding personalized treatment (stratified based on T-cell cytotoxicity intensity and Treg conversion degree, such as mTOR inhibitor combined with TSHR targeted therapy), identifying potential therapeutic targets (e.g., targeting the IL-15-STAT5 axis or the CXCR6-CXCL16 axis).	(96, 98–100)
pSS	scRNA-seq combined with scTCR/BCR-seq	Peripheral blood, labial gland	B cells exhibit IGHV gene selection bias; TCR diversity is reduced; CD8^+^ tissue-resident memory T cells serve as the core effector cells in glandular injury.	Disease classification (e.g., IFN-I dominant, cytotoxic T cell-enriched, TGF-β-driven), guiding precision immunotherapy interventions (e.g., JAK inhibitors for IFN-I type, CXCR6 targeting to block TRM migration), and prognosis assessment (glandular clonal expansion correlates with clinical activity).	(102, 103, 105, 107)
MS	scRNA-seq combined with scTCR/BCR-seq	Cerebrospinal fluid, peripheral blood	Local activation of B cells in cerebrospinal fluid; oligoclonal expansion of cytotoxic CD4^+^ T cells and CD8^+^ TEMRA cells; associated with EBV antigens.	Diagnosis and subtype differentiation (e.g., distinguishing RRMS from SPMS via CD8^+^ TEMRA), treatment guidance (e.g., anti-CD20 monoclonal antibodies for pathogenic B cell depletion, T-bet inhibitors targeting CD8^+^ TEMRA), and prognosis assessment (specific clonal frequency correlates with disability severity and progression rate).	(111–114)
T1D	scRNA-seq combined with scTCR/BCR-seq	Peripheral blood, pancreas	Islet antigen-reactive B cells show oligoclonal expansion; common TCR clones are present in CD4^+^ T cells; cytotoxic CD8^+^ T cells infiltrate the pancreas.	Early warning and disease staging (e.g., monitoring IAR B-cell clonal frequency), guiding stage-appropriate treatment (e.g., targeting common TCR clones during initial phase, suppressing CD8^+^ T-cell cytotoxicity during diagnosis phase), predicting treatment response and risk (e.g., pro-inflammatory memory CD4^+^ T-cell frequency predicting resistance to CD2-targeted drugs).	(119–121, 139)
Psoriasis	scRNA-seq combined with scTCR/BCR-seq	Skin, peripheral blood, joint synovial fluid	IL-26+ TH17 intermediate cells and Th17/Tc17 cell clonal expansion; CLA⁻ T cells mediate systemic inflammation.	Disease Subtyping and Progression Risk Assessment (e.g., CLA⁻ T cell frequency >3.3% indicates systemic inflammatory subtype and high PsA risk), Guiding Precision Therapy (IL-26 neutralizing antibodies for cutaneous-limited type, JAK inhibitors for systemic type, IL-17 inhibitors for PsA), Treatment Response Markers (Th17/Tc17 signature gene expression changes).	(125–127, 140)
PV	scRNA-seq and TCR sequencing	Dermatological lesion tissue	Antibody-secreting cells undergo oligoclonal expansion with high levels of somatic hypermutation; CXCL13^+^ CD4^+^ T cells exhibit clonal expansion; CD8^+^ TRM cells mediate cytotoxicity.	Uncovering mechanisms sustaining chronic inflammation to guide targeted therapies (e.g., rituximab depletion of CD20^+^ B-cell precursors, IL-21R antagonists or CCR6 inhibitors targeting the TLS niche), and monitoring treatment response (peripheral blood CXCL13 levels indicate TLS activity).	(133–135, 137)
AIHA	scTCR/BCR-seq	bone marrow	CD8^+^ T cell clones undergo dynamic changes: initial clones are hormone-sensitive, while cytotoxic clones correlate with disease chronicity.	Guiding treatment decisions (use of JAK inhibitors during early expansion of cytotoxic CD8^+^ T cell clones), predicting prognosis (clonal dynamics correlate with chronic/relapsed disease), and revealing resistance mechanisms (initial clones exhibit hormone sensitivity, while cytotoxic clones drive persistent disease activity).	(141)
AIH	scTCR/BCR-seq	Liver, peripheral blood	Secretes IFN-γ/TNF and assists B cells via IL-21.	Guide targeted therapies (such as IL-21/IFN-γ bispecific antibodies or IL-21R blockers), monitor disease activity (serum IL-21 levels >35 pg/mL indicate excessive B-cell activation), and reveal persistent immune abnormalities during remission (persistent presence of SLA-specific T cells).	(142)
AS	scTCR/BCR-seq	Synovial fluid, peripheral blood	Active phase effect: T/B cell oligoclonal expansion; Th17 and Treg cells express dual TCRs with impaired function.	Assessment of disease activity (clonal expansion and diversity changes), identification of novel therapeutic targets (e.g., TRAIL antibody neutralization of dual TCR pTh17 cells, histone deacetylase inhibitors restoring Treg function), and understanding of immune plasticity (TCR sharing among T cell subsets).	(143, 144)

The same analytical framework that has produced these insights is now being applied to a broader spectrum of autoimmune disorders with more complex or less well−defined etiologies, underscoring their versatility. In autoimmune hemolytic anemia, longitudinal scTCR−seq has dynamically traced the evolution of T−cell clones from active disease to remission, directly linking clonally expanded cytotoxic CD8^+^ T cells to impaired erythropoiesis in the bone marrow (141). In autoimmune hepatitis, a population of dual−function CD4^+^ T−cell clones were identified within the liver; these clones contribute to hepatocellular injury via cytokine production while also helping B cells generate autoantibodies, offering a new perspective on the sustained autoimmune attack in the liver (142). In ankylosing spondylitis, analysis of affected synovial tissue not only confirmed oligoclonal expansion of effector T cells but, more importantly, revealed clonal−level connections between a uniquely pathogenic Th17 subset and dysfunctional regulatory T cells, highlighting both the complexity of the local inflammatory milieu and the plasticity of T−cell phenotypes (143, 144).

The methodological value of integrating immune cell clonality, functional subpopulations, and spatial localization via single-cell technologies—emphasized in this review—has been preliminarily and robustly validated across diverse disease models. As the field advances, systematic cross−disease comparisons of the kind initiated here will be crucial for extracting generalizable principles from the rapidly expanding single−cell immune repertoire datasets in autoimmunity.

## 4 Technical challenges and future perspectives

While scTCR/BCR-seq has driven major advances in autoimmune disease research, translating these discoveries into routine practice still faces several core challenges. These hurdles occur at three levels: technical sensitivity and completeness, multi-dimensional data integration, and clinical translation pathways.

### 4.1 Technical and interpretive hurdles

At the technical level, the limited detection of low abundance pathogenic clones remains a primary constraint. Insufficient sequencing depth, susceptibility of RNA samples to degradation, and inevitable chain loss during single-cell capture and amplification can lead to the omission of critical low-frequency clones or paired receptor information. Consequently, the diversity of immune receptor repertoire and its clonal architecture may be profiled in a biased manner. This limitation directly undermines the completeness of clonal tracking, particularly for rare but functionally important populations. While robust computational frameworks now exist for many of these tasks (Section 1.4), their application across diverse datasets generated from different platforms and centers remains a persistent bottleneck. Closely related to the technical challenge is the difficulty of integrating scTCR/BCR data with other single cell modalities. A persistent bottleneck is the unbiased consolidation of TCR/BCR sequences with single-cell transcriptomic, epigenomic, and proteomic data derived from disparate platforms. Batch effects and technical noise severely complicate the accurate reconstruction of a unified cellular identity for individual immune cells, one that simultaneously defines clonotype, molecular phenotype, and functional state. Thus, even very large datasets may yield limited mechanistic insight.

A further limitation is the field’s over-reliance on correlative data. Identifying a clonally expanded population in diseased tissue is suggestive but does not prove pathogenicity. A crucial gap remains between clone discovery and functional validation. Only a minority of studies demonstrate antigen specificity of identified TCRs/BCRs or use adoptive transfer to establish pathogenic potential. Future work should prioritize such validation to move from descriptive associations to causal mechanisms.

### 4.2 The path to clinical translation: beyond clone identification

While scTCR/BCR-seq excels at identifying expanded clones and linking them to disease, clinical translation requires progress beyond clone discovery. A primary translational bottleneck lies in the functional validation gap. As noted earlier, sequencing can pinpoint clonally expanded populations, but definitive proof of their pathogenic role still heavily relies on traditional, low-throughput experimental models—such as antigen-specific T-cell assays or transgenic animals—which are time-consuming and difficult to scale. This disconnect between sequence identification and mechanistic confirmation delays the translation of findings into validated therapeutic targets.

Even with robust functional data, significant pharmacological and developmental barriers impede the creation of clonally targeted therapies. Achieving the precision needed to eliminate specific TCR or BCR clonotypes without broad immunosuppression is formidable. Patient-specific approaches such as CAR-T may, in principle, provide such specificity, but their application to autoimmunity faces high costs, regulatory complexity, and manufacturing hurdles—barriers that are particularly acute for personalized treatments.

In parallel, clonotype-derived biomarkers must be advanced toward clinical utility. Transforming research observations—for example, the expansion frequency of a given clone—into validated prognostic or predictive biomarkers requires rigorous, standardized qualification in large, prospective cohorts to establish analytical robustness, sensitivity, specificity, and clinical value. Currently, the lack of standardized protocols for sample processing, data analysis, and cross-institutional interpretation impedes biomarker development and broader deployment.

Underlying these translational issues are persistent technical and analytical constraints that limit real-world use. High sequencing costs, batch effects, challenges in multi-omics integration, and the absence of unified analytical frameworks restrict scTCR/BCR-seq in large-scale screening and multicenter studies. Together, these barriers slow the transition from research insight to routine clinical implementation.

Therefore, realizing the full potential of single-cell immune receptor sequencing in autoimmune disease management will require a coordinated effort to bridge functional validation gaps, develop practical and selective therapeutic modalities, establish rigorous biomarker development pathways, and address the technical and infrastructural barriers that constrain clinical adoption.

### 4.3 Future perspectives: methodological innovation and translational breakthroughs

To elevate single-cell technologies from exploratory instruments to pivotal diagnostic and therapeutic modalities, future endeavors must systematically address current methodological constraints. Foundational methodological refinements are paramount. Specifically, progress hinges upon optimizing single-cell capture technologies to achieve higher sensitivity and minimize chain loss, coupled with long-read sequencing platforms (e.g., Pacific Biosciences or Oxford Nanopore). Emerging commercial platforms (e.g., 10x Genomics GEM-X, Scale Bio, Parse Biosciences) promise higher throughput and improved chain pairing recovery through combinatorial indexing or optimized microfluidics. Such integration facilitates the precise elucidation of full-length TCR/BCR sequences and their somatic hypermutation patterns. These advances should increase information yield and, over time, reduce per-sample costs, easing major technical and economic barriers to routine clinical use.

Concurrently, unified platforms for simultaneous multi-omics profiling are needed, coupled with specialized bioinformatics algorithms tailored to autoimmunity. Key goals include automated detection of expanded clones, reliable prediction of TCR/BCR antigen specificity, and the creation of standardized, shareable immune receptor repertoire reference databases. Such computational advances are essential to maximize scientific and clinical utility. In parallel, the integration of scTCR/BCR-seq with spatial transcriptomics will enable the mapping of specific clonotypes to their histopathological niches, directly linking clonal activity to tissue microanatomy. Bridging to clinical application also requires expanding translational pathways and re-engineering functional validation systems. Near-term opportunities include non-invasive diagnostics and dynamic disease monitoring anchored to clonotype-specific sequences. Longer-term aims envision precision therapies that target pathogenic clones (e.g., CAR-T, TCR-T, or personalized vaccines). To accelerate this pipeline, validation paradigms should incorporate single-cell CRISPR screening, patient-derived organoids, and high-throughput antigen-specific assays, enabling rapid and physiologically relevant testing of candidate clones and shortening the path from sequence discovery to target confirmation.

## 5 Conclusions

This review synthesizes recent advances in integrating single-cell immune repertoire sequencing (scTCR/BCR-seq) with scRNA-seq to elucidate pathogenic mechanisms in autoimmune diseases. Across ten major disorders, these approaches delineate clonal expansion, tissue infiltration, and functional dysregulation of autoreactive T and B cells. Joint analysis of repertoire and transcriptomic data reveals disease-specific clonal architectures, pathogenic subpopulations (e.g., age-associated B cells, tissue-resident memory T cells), and self-amplifying inflammatory loops, establishing a framework for precision subtyping and rational target selection.

Looking ahead, continued improvements in experimental workflows, multi-omics integration, and functional validation are poised to address current limitations of scTCR/BCR-seq. These developments will clarify core pathogenic pathways and enable diagnostic and therapeutic strategies grounded in clonal signatures, supporting patient stratification and individualized intervention. By shifting clinical practice from broad immunosuppression to mechanism-based, clone-directed treatment, single-cell repertoire profiling has the potential to deliver more durable and safer outcomes for patients.
